# Assessing health system performance

**DOI:** 10.2471/BLT.24.020724

**Published:** 2024-07-01

## Abstract

The post-pandemic era presents an opportunity to prioritize health system performance assessment. Adèle Sulcas reports.

What Professor Mushtaque Chowdhury most remembers about the first weeks of Bangladesh’s COVID-19 epidemic was the response of the public health authorities.

“We were basically not ready,” recalls the Founding Dean of the School of Public Health at BRAC University, Dhaka. “First, there was a general sense of denial, and when a response effort was finally initiated, screening was inadequate as were quarantine and lockdown measures, while there was insufficient personal protective equipment for health-care providers.” Chowdhury also points to “inadequate communication regarding the risks of transmission.”

By mid-July 2020, the authorities had reported 230 000 cases, and systemic inadequacies had been exposed, notably a lack of primary health-care capacity (including a capacity to source, distribute and administer medical grade oxygen) and what Chowdhury considers “a complete lack of coordination between key institutions.”

Needless to say, Bangladesh was not the only country to struggle in the face of COVID-19. “The pandemic exposed the lack of resilience in health systems worldwide,” says Dheepa Rajan, a health systems specialist at the European Observatory of Health Systems and Policies, a hosted partnership of the World Health Organization (WHO), based in Brussels.

For Rajan, while clearly cause for concern, that exposure has also created conditions in which governments are paying greater attention to the challenges their health systems confront in the face of emergencies. 

Not for the first time. As Rajan points out, a comparable period of reflection followed the outbreak of severe acute respiratory syndrome coronavirus in 2002–2003, which also exposed health system resilience issues.

It was partly that exposure that led to the drafting of the 2005 *International health regulations *(IHR), which was accompanied – as Rajan also points out – by a tool for States Parties Self-Assessment Annual Reports (SPAR).

“The pandemic exposed the lack of resilience in health systems.”

Dheepa Rajan

SPAR were designed to help health authorities assess their systems’ capacity to detect, report on and respond to health emergencies. However, though laudable in intention, SPAR have faced challenges ranging from self-assessment bias (a tendency to under-count and under-report weakness) to lack of resources required to carry out assessment, including functioning health information systems.

Since 2016, WHO has been supporting countries with assessment focused on health emergency prevention, detection and response through voluntary, collaborative evaluation exercises known as Joint External Evaluations (JEEs). Over 100 countries have undertaken JEEs since 2016, but few have translated assessment findings into policy action.

Bangladesh is a case in point. The country was the fifth to complete the JEE process in 2016, and among its findings was weakness in coordination between and within ministries – a weakness which, according to Chowdhury, continues to be a problem.

Viet Nam also undertook a JEE in 2016. According to Angela Pratt, WHO Representative in Viet Nam since 2022, it helped the authorities identify critical gaps in its health system and generated 74 recommendations, including recommendations for emergency response.

The latter were eventually fed into a draft national master plan in 2019. However, according to Pratt, the plan was never approved by the government because the COVID-19 pandemic hit soon after. She notes, nevertheless, that the plan did form an important input into how the country responded to COVID-19.

For Pratt, the Viet Nam experience highlights the fact that assessments take place in evolving political and economic contexts in which governments must weigh different priorities. “Health system assessment does not automatically lead to a national plan and then to a set of improvements,” she says. “The political and policy context is usually more complex than that.” According to Pratt, a second JEE is expected towards the end of 2024, when she hopes that health system issues raised during the pandemic will be addressed, including outbreak surveillance capacity.

Rajan would like to see more JEEs coordinated with full health system performance assessments, and vice versa. “Ideally, health system performance assessments and evaluations of health emergency preparedness come together in a coordinated way with efforts to assess health system performance as a whole,” she explains.

WHO has been encouraging such comprehensive assessment since the publication of the Health Systems Performance Assessment (HSPA) framework that was released as part of the 2000 world health report, *Health systems: improving performance*.

Rajan is hopeful that a new WHO HSPA framework, will help draw attention to the issue while providing essential guidance.

The 2022 framework includes the four original cornerstone ‘functions’ – governance, resource generation, financing and service delivery – along with their many sub-functions, as well as intermediate objectives (quality and access) and final goals (health improvement, financial protection and people-centredness).

Crucially, the new framework emphasizes connections across systems. “Assessment efforts in countries too often take place in a piecemeal way with a narrow focus on single issues, such as childhood vaccinations or disease-specific programme performance,” says Rajan. “This not only encourages silo-oriented thinking, it fails to capture the vital connections across the system which impact performance just as strongly as individual components do.”

Cross-sectoral assessment also needs to be embraced, in Rajan’s view. “Comprehensive HSPA should provide a sense of how well health is working with other sectors, since so many of today’s challenges within health are linked to things happening outside of it,” she says.

Finally, Rajan is keen to see assessment capturing qualitative variables such as patient satisfaction as much as quantitative variables like patient beds.

That such an approach yields benefits is already being demonstrated. In Belgium, for example, after extensive preparation and consultation, the health authorities started this type of health system assessment in 2009 and, to date, have produced five comprehensive HSPA reports and six interim reports.

According to Pascal Meeus, a public health and health data management specialist who was instrumental in getting the Belgian health authorities to adopt HSPA, the initial intention was to determine whether the government was getting value for money. The scope of assessment subsequently expanded.

“Before the HSPA era in Belgium, as in other European Union countries, reports on the health system were mostly oriented towards costs,” he explains. “Now we have a balance with other dimensions such as accessibility, quality, safety, equity, sustainability and resilience which helps to align stakeholders on common objectives.”

“We were basically not ready.”

Mushtaque Chowdhury

Meeus emphasizes that it is not just a question of capturing a greater variety of information but of integrating the information to achieve a more comprehensive, holistic and actionable diagnosis.

Rajan would like to see others reap those benefits, but she is clear-eyed about the challenges faced, the first being a lack of agreement on what exactly HSPA is – a lack that explains, in part, the slow development and uptake of HSPA.

“The concept and practice of HSPA has evolved, with differing interpretations and methodologies,” she says, noting the efforts of the Organisation for Economic Co-operation and Development, the United States Agency for International Development (USAID) and the World Bank, and calling on the different actors to work towards harmonization.

The paucity of health information captured in many countries is another major challenge but, as Rajan is quick to point out, many countries have made great strides in this regard.

Rwanda is a prime example. Since 2006, the government has focused on monitoring and surveillance as part of the regular performance-based contracting procedures implemented under its Imihigo system – a national health systems strengthening project aimed at enhancing financial protection and access to quality health-care services.

According to Solange Hakiba, until recently Chief of Party for the USAID–Rwanda Integrated Health Systems Activity, the Imihigo system was developed to monitor the performance of public services with a view to improving them. “The Imihigo system has not only enhanced data capture,” she says, “it has helped instil a culture of accountability and results-oriented management within the public sector.”

Rwanda conducted its first JEE in 2018 to assess the country’s capacity to implement IHR (2005). The JEE was multisectoral, identifying strengths and gaps in various technical areas essential for managing public health threats, including disease surveillance, response systems and laboratory capacities​. According to Hakiba, progress has since been made in each of these areas. “We are in a region where outbreaks and other health emergencies can occur at any time,” she says. “We have to be ready.”

A final major challenge to broader uptake of HSPA is a reluctance to engage in what can seem like a daunting exercise. Even in countries with the capacity to carry out HSPA, adoption has not always been straightforward. In Belgium, for example, sceptics had to be convinced. “People didn’t necessarily see the value of health system assessment as inputs into policy, and they doubted its sustainability,” says Meeus. “That has changed.”

Rajan would like to see others come to the same realization. “What we’d really like to see is a solid process of defining the objectives collectively with all the relevant stakeholders, orienting the actual assessment to be included into the policy cycle,” she says. “Inclusivity is key to better policy uptake, and crucial to better implementation and impact on the ground.”

**Figure:**
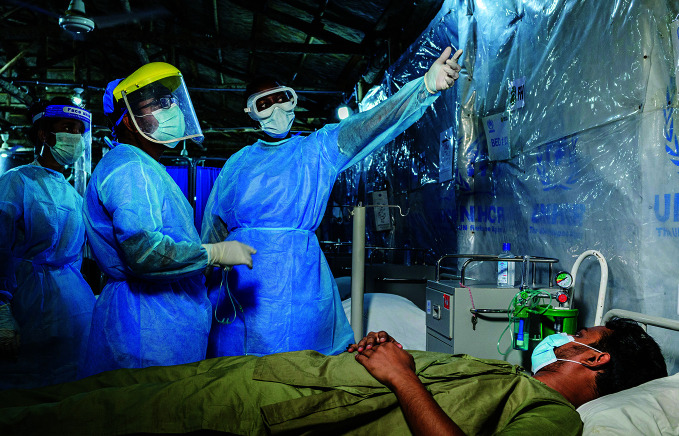
An infection prevention and control specialist inspects an isolation centre in Cox's Bazar, Bangladesh

WHO/Blink Media - Fabeha Monir

